# A functional variant of the dimethylarginine dimethylaminohydrolase-2 gene is associated with myocardial infarction in type 2 diabetic patients

**DOI:** 10.1186/s12933-019-0906-1

**Published:** 2019-08-13

**Authors:** Gaia Chiara Mannino, Serena Pezzilli, Carolina Averta, Anastasia Fuoco, Rosangela Spiga, Elettra Mancuso, Concetta Di Fatta, Francesco Perticone, Sabrina Prudente, Vincenzo Trischitta, Francesco Andreozzi, Giorgio Sesti

**Affiliations:** 1 Department of Medical and Surgical Sciences, University “Magna Graecia” of Catanzaro, Viale Europa, 88100 Catanzaro, Italy; 2 Research Unit of Metabolic and Cardiovascular Diseases, Fondazione IRCCS Casa Sollievo della Sofferenza, San Giovanni Rotondo, Italy; 3 Medical Genetics, University Chieti-Pescara, Pescara, Chieti Scalo Italy; 4 Department of Experimental Medicine, Sapienza University, Rome, Italy

**Keywords:** Asymmetric dimethylarginine, Myocardial infarction, Dimethylarginine dimethylaminohydrolase, Type 2 diabetes, rs9267551

## Abstract

**Background:**

Myocardial infarction is the main mortality cause in patients with type 2 diabetes (T2DM). Endothelial dysfunction due to reduced bioavailability of nitric oxide (NO) is an early step of atherogenesis. Asymmetric dimethylarginine (ADMA) is an endogenous inhibitor of NO synthesis, and it is metabolized by the enzymes dimethylarginine dimethylaminohydrolase (DDAH) 1 and 2. The functional variant rs9267551 C, in the promoter region of *DDAH2*, has been linked to increased *DDAH2* expression, and lower ADMA plasma levels, and was associated with lower risk of coronary artery disease in large-scale genome-wide association studies (GWAS) performed in the general population. However, it is unknown whether this association holds true in T2DM patients. To address this issue, we investigated whether rs9267551 is associated with risk of myocardial infarction in two cohorts of T2DM patients.

**Methods:**

SNP rs9267551 was genotyped in 1839 White T2DM patients from the Catanzaro Study (CZ, n = 1060) and the Gargano Heart Study-cross sectional design (GHS, n = 779). Cases were patients with a previous myocardial infarction, controls were asymptomatic patients with neither previous myocardial ischemia nor signs of it at resting and during a maximal symptom limited stress electrocardiogram.

**Results:**

Carriers of allele rs9267551 C showed a dose dependent reduction in the risk of myocardial infarction [(CZ = OR 0.380, 95% CI 0.175–0.823, *p *= 0.014), (GHS = 0.497, 0.267–0.923, *p *= 0.027), (Pooled = 0.458, 0.283–0.739, *p *= 0.001)] which remained significant after adjusting for sex, age, BMI, smoking, HbA1c, total cholesterol HDL, and triglyceride levels [(CZ = 0.307, 0.106–0.885, *p *= 0.029), (GHS = 0.512, 0.270–0.970, *p *= 0.040), (Pooled = 0.458, 0.266–0.787, *p *= 0.005)].

**Conclusions:**

We found that rs9267551 polymorphism is significantly associated with myocardial infarction in T2DM patients of European ancestry from two independent cohorts. It is possible that in subjects carrying the protective C allele less ADMA accumulates in endothelial cells causing vascular protection as a consequence of higher nitric oxide availability.

## Background

Cardiovascular disease (CVD) is a main cause of morbidity and mortality among people with type 2 diabetes (T2DM), and myocardial infarction (MI) remains the most common cause of mortality in these patients [1–3]. Indeed, the presence of diabetes doubles the risk of mortality in individuals with history of myocardial infarction [4]. Endothelial dysfunction due to reduced bioavailability of nitric oxide (NO) is an early step in the atherogenesis. NO is synthesized by the endothelial NO synthase (eNOS), whose activity is negatively regulated by the endogenous inhibitor asymmetric dimethylarginine (ADMA), a naturally occurring methylated arginine competing with l-arginine as a substrate for eNOS [5, 6]. Although elevated plasma levels of ADMA have been associated with major cardiovascular events and mortality in patients with different cardiovascular risk profile [7–11], the role of ADMA in predicting CVD among patients with diabetes is still subject of debate [12–15]. ADMA is endogenously generated by several protein arginine methyltransferases (PRMTs), and is metabolized by the enzyme dimethylarginine dimethylaminohydrolase (DDAH) [16, 17], which is encoded by two distinct genes: *DDAH1* and *DDAH2*. DDAH1 is localized in tissues expressing neuronal NOS, whereas DDAH2 is highly expressed in the endothelium [18]. Previous studies assessing the association of *DDAH2* polymorphisms with the risk of CVD have led to conflicting results [19–21]. Amongst the *DDAH2* polymorphisms, the rs9267551 G/C variant in the promoter region of *DDAH2* has been demonstrated to have a functional impact with the C allele showing increased transcriptional activity and higher expression of DDAH2 transcript in primary human endothelial cells naturally carrying the C allele [22]. In addition, adult carriers of the rs9267551 C allele exhibited lower circulating levels of ADMA, higher insulin sensitivity, and lower risk of chronic kidney disease [22]. To explore the relationship between this functional variant and CVD, we interrogated the PhenoScanner database holding publicly available results from large-scale genome-wide association studies (GWAS) [23] (http://www.phenoscanner.medschl.cam.ac.uk/). The variant rs9267551 C was associated ([24] OR = 0.95, 95% CI 1.028–1.085; p = 3.88 × 10^−8^) with lower risk of coronary artery disease (CAD), defined as fatal or nonfatal MI, percutaneous transluminal coronary angioplasty or coronary artery bypass grafting, chronic ischemic heart disease and angina. However, it is unclear whether this putative association holds true even in patients with T2DM. In view of the important role of ADMA in regulating endothelium dependent vasodilation and predicting cardiovascular events and mortality in the general population, we investigated whether the functional polymorphism rs9267551 in the promoter region of *DDAH2* is associated with risk of MI in T2DM patients too. To this aim, we investigated the association of this polymorphism with MI in two independent cohorts of T2DM patients of European ancestry.

## Materials and methods

### Study participants

This study comprises 1060 patients with T2DM from the Catanzaro Study and 779 participants from the Gargano Heart Study-cross sectional design (GHS) [25]. In both studies T2DM was defined according to the American Diabetes Association (ADA) criteria [26].

The Catanzaro Study is a cohort of White patients with T2DM recruited at the University Magna Graecia in Catanzaro, Italy. History of cardiovascular events such as acute myocardial infarction, angiographic evidence of stenosis > 50% in at least one major coronary artery or their main branches, smoking, hypertension, hypercholesterolemia and treatment with glucose-lowering drugs were self-reported at the time of examination, and confirmed by review of medical records. Exclusion criteria were presence of end stage renal disease, chronic gastrointestinal diseases associated with malabsorption, chronic pancreatitis, history of any malignant disease, self-reporting alcohol consumption > 20 g/day, positivity for antibodies to hepatitis C virus (HCV) or hepatitis B surface antigen (HBsAg).

In the original case–control design of GHS, cases were patients with previous myocardial infarction or with coronary a stenosis > 50% in at least one coronary major vessel at angiography. Controls were asymptomatic patients without signs of myocardial ischemia at resting and during a maximal symptom limited stress electrocardiogram (ECG) or with stenosis < 50% at coronary angiography [27]. For this specific study, cases being as such because of coronary stenosis with no history of myocardial infarction, were considered as controls. For this reason, presence/absence of coronary stenosis in controls was used as a binary variable to be accounted for in multivariate logistic regression analysis (see statistical methods). The only exclusion criterion was the presence of poor life expectancy due to non–diabetes-related disorders, such as severe infectious illnesses or any type of cancer.

In all, in both samples, cases were patients with a previous myocardial infarction whereas controls were asymptomatic patients with neither previous myocardial ischemia nor signs of it at resting and during a maximal symptom limited stress ECG.

The study protocol and informed consent procedures were approved by the local research ethics committees (Comitato Etico Azienda Ospedaliera “Mater Domini”, Catanzaro (Italy); and IRCSS “Casa Sollievo della Sofferenza”, San Giovanni Rotondo (Italy)). This study was conducted in accordance with the principles of the Declaration of Helsinki and all subjects provided signed informed consent before commencing the studies.

### Measurements and analytical determinations

All participants underwent anthropometrical evaluation including measurements of body mass index (BMI). In the Catanzaro Study, plasma glucose, triglyceride, total and high density lipoprotein (HDL-C) cholesterol concentrations were determined by enzymatic-colorimetric methods using a Roche Cobas^®^ 4000 analyzer, while glycated hemoglobin (HbA1c) was assessed by high performance liquid chromatography using a National Glycohemoglobin Standardization Program (NGSP) certified automated analyzer (Adams HA-8160 HbA1C analyzer, Menarini, Italy).

In the GHS, plasma glucose and lipid profile (total serum cholesterol, HDL cholesterol, and serum triglycerides) were determined by a Roche Modular P Chemistry Analyzer, while HbA1c was determined by high-pressure liquid chromatography after removal of the labile fraction (HPLC Diamat Analyzer; Bio-Rad, Richmond, CA).

### Genotyping of DDAH2 gene polymorphism

Human blood samples were collected from all patients. DNA was extracted from whole blood using commercial DNA isolation kits (Promega, Madison, WI and Roche, Mannheim, Germany). rs9267551 DDAH2 genotype calls were determined with TaqMan allelic discrimination assay (Assay ID# C__27848488_10; Applied Biosystems, Foster City, CA). For the Catanzaro Study template DNA was amplified and fluorescence was detected on an iCycler Thermal Cycler with iQ5 Multicolor Real-Time PCR Detection System (Bio-Rad Laboratories, Inc., Hercules, CA). Good genotyping quality was ensured by including 0.05 ng of custom oligo strings (GeneArt^®^ Strings™ DNA Fragments, Invitrogen, Thermo Fisher Scientific) with a sequence designed to span symmetrically ~ 200 bp around the context sequence of the genotyping assay, differing only for the rs9267551 allele C or G. The oligo strings were combined and loaded as three individual samples representing one heterozygous C/G and two sets of homozygous C/C and G/G controls, in each 96-well plate. Genotyping concordance of the oligo strings was 100%. The GHS genotyping was performed on a HT 7900 platform (Applied Biosystems, Foster City, CA, USA). Genotyping quality was tested by including six blinded duplicate samples in each 384-well assay. The average agreement rate of duplicate samples was > 99%.

### Statistical analysis

Clinical patients’ characteristics are reported as mean ± standard deviation (SD) for continuous data (SD). Unpaired Student’s test was used to compare differences of continuous variables between two groups, and the *χ*^2^-test for non-continuous variables. Log transformation was used when analyzing triglycerides levels because their distribution did not respect the assumption of normality. Therapies were coded as binary variables where 0 indicated absence of pharmacological treatment, 1 meant use of oral or injectable hypoglycemic agents for the category of glucose lowering therapy; use of sartans, angiotensin converting enzyme inhibitors, beta-blockers, Ca^2+^ antagonists, or diuretics, in combination or alone, as anti-hypertensive therapy; use of statins as anti-dyslipidemic therapy. A 0–1 binary variable was also used to identify among GHS controls those who without or with coronary stenosis. The Hardy–Weinberg equilibrium between the genotypes was evaluated by *χ*^2^ test. A multivariate logistic regression analysis was employed to test the association between the rs9267551 polymorphism and MI and results were reported as odds ratios (ORs) with 95% CIs. All tests were two-sided, and a *p* value < 0.05 was considered statistically significant. All calculations were done with SPSS software program Version 22.0 for Windows. We have determined that the case–control sample had 86% power (α = 0.05) for detecting a risk allele with an odds ratio (OR) of 1.6 for MI assuming an additive genetic model.

## Results

Clinical features of case and control subjects from the two studies are shown in Table 1. In total, 1839 patients affected by T2DM were recruited from two independent studies and stratified according to the presence of MI (Table 1).

**Table 1:** Clinical features of the study subjects

Characteristics	CZ			GHS		
	MI-neg	MI-pos	*p*	MI-neg	MI-pos	*p*
*n*	883	177		595	184	
Males (%)	57.0	75.7	< 0.001	50.4	71.7	< 0.001
Age (years)	61.0 ± 11.5	64.8 ± 9.2	< 0.001	61.2 ± 8.6	64.2 ± 8.5	< 0.001
Age at diabetes diagnosis (years)	53 ± 12	54 ± 11	0.65	49 ± 10	50 ± 11	0.43
Diabetes duration (years)	7.9 ± 8.7	11.2 ± 9.7	< 0.001	11.8 ± 8.4	14.1 ± 9.6	< 0.01
BMI (kg/m^2^)	31.3 ± 6.4	30.1 ± 4.9	< 0.02	30.9 ± 5.2	30.8 ± 4.8	0.56
HbA1c (%)	7.49 ± 1.64	7.74 ± 1.80	0.13	8.57 ± 1.89	8.61 ± 1.81	0.81
Total cholesterol (mg/dl)	190.9 ± 43.6	170.6 ± 42.9	< 0.001	192.2 ± 46.6	173.3 ± 46.2	< 0.001
HDL cholesterol (mg/dl)	46.7 ± 14.2	44.1 ± 11.9	0.02	45.1 ± 12.5	44.1 ± 15.9	0.37
Triglycerides (mg/dl)	157.9 ± 99.9	156.4 ± 95.5	0.84	152.8 ± 100.1	159.4 ± 102.4	0.74
Glucose-lowering therapy (%)	63.3	79.1	< 0.001	83.7	85.9	0.48
Anti-hypertensive therapy (%)	70.1	90.4	< 0.001	70.8	88.6	< 0.001
Anti-dyslipidemic therapy (%)	34.7	70.6	< 0.001	40.3	73.9	< 0.001
Ever smoked (%)	44.8	64.4	< 0.001	33.9	44.0	< 0.02

*CZ* Catanzaro Study, *GHS* Gargano Heart Study, *MI* myocardial infarction, *BMI* body mass index, *HbA1c* glycated hemoglobin

In the Catanzaro Study, 177 patients had MI and 883 patients were MI-negative controls, while in the GHS, 184 patients had MI and 595 patients were MI-negative controls. In both studies, patients with previous MI were older, more likely to be males, and longer duration of T2DM as compared with MI-negative controls (Table 1). Total cholesterol levels were significantly lower in MI-positive patients, likely attributable to the highest percentage of anti-dyslipidemic therapies. In the Catanzaro Study, BMI and HDL levels were significantly lower among MI-positive cases as compared with controls. In addition, more MI-positive patients were treated with glucose-lowering agents.

The genotype distribution was consistent with the Hardy–Weinberg equilibrium (p > 0.1) in both the GHS and the Catanzaro Study. No evidence of SNP-by-study sample interaction was observed (p = 0.22).

No significant differences among the three genotypes were observed in relation to age, gender, smoking habit, BMI, lipid profile, age at the diagnosis of T2DM, duration of diabetes, and treatment with anti-dyslipidemic, anti-hypertensive or anti-diabetic drugs, with the exception of HbA1c levels in the Catanzaro Study which were significantly higher in carriers of the C allele (Table 2).

**Table 2:** Clinical characteristics of 1839 study subjects according to the SNP rs9267551

Characteristics	CZ				GHS			
	*GG*	*GC*	*CC*	p	*GG*	*GC*	*CC*	p
*n*	966	90	4		694	82	3	
Males (%)	60.8	54.4	25.0	0.18	55.0	57.3	100.0	0.27
Age (years)	61.6 ± 11.2	62.1 ± 11.7	57.5 ± 12.7	0.73	62 ± 9	60 ± 9	68 ± 14	0.09
Age at diabetes diagnosis (years)	53 ± 12	55 ± 13	50 ± 6	0.35	49 ± 10	48 ± 9	53 ± 13	0.59
Diabetes duration (years)	8.6 ± 9.0	7.2 ± 8.8	8.0 ± 7.7	0.37	12.4 ± 8.8	11.9 ± 8.8	15.3 ± 5.0	0.74
BMI (kg/m^2^)	31.0 ± 6.1	31.2 ± 6.9	33.7 ± 5.8	0.65	30.8 ± 5.1	31.7 ± 5.3	31.2 ± 3.7	0.34
HbA1c (%)	7.48 ± 1.63	8.03 ± 1.92	8.16 ± 1.92	0.03	8.57 ± 1.88	8.68 ± 1.87	9.10 ± 2.38	0.79
Total cholesterol (mg/dl)	186.9 ± 44.3	192.3 ± 42.0	213.7 ± 43.2	0.26	188.4 ± 47.7	182.7 ± 41.6	181.3 ± 68.6	0.56
HDL cholesterol (mg/dl)	46.1 ± 13.9	47.3 ± 12.9	52.2 ± 13.8	0.51	44.9 ± 13.6	44.6 ± 12.2	43.0 ± 6.1	0.95
Triglycerides (mg/dl)	158.1 ± 95.4	156.7 ± 133.6	105.7 ± 40.9	0.43	155.1 ± 101.5	149.5 ± 93.1	126.7 ± 92.4	0.70
Glucose-lowering therapy (%)	65.7	68.9	50.0	0.66	84.1	84.1	100.0	0.75
Anti-hypertensive therapy (%)	74.1	66.7	75.0	0.31	73.8	82.8	66.7	0.19
Anti-dyslipidemic therapy (%)	41.7	31.1	0.00	0.03	47.8	51.2	66.7	0.68
Ever smoked (%)	48.1	47.8	50.0	0.99	36.6	34.1	33.3	0.90

*CZ* Catanzaro Study, *GHS* Gargano Heart Study; *MI* myocardial infarction, *BMI* body mass index, *HbA1c* glycated hemoglobin

Carriers of the C allele showed a dose dependent decrease in the risk of MI in the Catanzaro Study (OR 0.380, 95% CI 0.175–0.823, p = 0.014) as compared with subjects carrying the GG genotype (Table 3).

**Table 3:** Association between rs9267551 and MI in the two study samples

Study samples	DDAH2 rs9267551 genotype			Additive model (unadjusted)		Additive model	
CZ (n = 1060)	GG	GC	CC	OR (95% CI)	p	OR (95% CI)	p
MI-neg (n = 883)	796 (90.1%)	83 (9.4%)	4 (0.5%)	0.380 (0.175–0.823)	0.014	0.342 (0.146–0.799)	0.013^a^
						0.307 (0.106–0.885)	0.029^b^
MI-pos (n = 177)	170 (96.0%)	7 (4.0%)	0 (0%)			0.364 (0.121–1.102)	0.074^c^

GHS (n = 779)	GG	GC	CC	OR (95% CI)	p	OR (95% CI)	p
M-neg (n = 595)	522 (87.7%)	70 (11.8%)	3 (0.5%)	0.497 (0.267–0.923)	0.027	0.503 (0.269–0.941)	0.031^a^
						0.512 (0.270–0.970)	0.040^b^
MI-pos (n = 184)	172 (93.5%)	12 (6.5%)	0 (0%)			0.478 (0.245–0.935)	0.031^c^

Pooled (n = 1839)	GG	GC	CC	OR (95% CI)	p	OR (95% CI)	p
MI-neg (n = 1478)	1318 (89.2%)	153 (10.3%)	7 (0.5%)	0.458 (0.283–0.739)	0.001	0.445 (0.270–0.733)	0.001^d^
						0.458 (0.266–0.787)	0.005^e^
MI-pos (n = 361)	342 (94.7%)	19 (5.3%)	0 (0%)			0.457 (0.259–0.808)	0.007^f^

*CZ* Catanzaro Study, *GHS* Gargano Heart Study

^a^Adjusted for sex, age, BMI, smoking habit

^b^Adjusted for sex, age, BMI, smoking habit, HbA1c, total cholesterol, HDL cholesterol and triglycerides levels

^c^Adjusted for sex, age, BMI, smoking habit, HbA1c, total cholesterol, HDL cholesterol, triglycerides levels, diabetes duration and therapies

^d^Adjusted for sex, age, BMI, smoking habit and study center

^e^Adjusted for sex, age, BMI, smoking habit, HbA1c, total cholesterol, HDL cholesterol, triglycerides levels and study center

^f^Adjusted for sex, age, BMI, smoking habit, HbA1c, total cholesterol, HDL cholesterol, triglycerides levels, diabetes duration, therapies and study center

To evaluate the impact of potential confounders on this association, we carried out a logistic regression analysis in a model adjusted for age, gender, BMI, and smoking habit. The association of the C allele with lower risk of MI remained significant after adjusting for sex, age, BMI, and smoking habit (OR 0.342, 95% CI 0.146–0.799 p = 0.013). Similarly, the association remained significant after adjusting for additional confounders including HbA1c, total cholesterol HDL cholesterol, and triglycerides (OR 0.307, 95% CI 0.106–0.885, p = 0.029). Though no longer significant, no much change was observed when treatments and duration of diabetes were further added to the logistic regression model (OR 0.364, 95% CI 0.121–1.102, p = 0.074).

A significant association between the C allele and lower risk of MI was also observed in the GHS cohort (OR 0.497, 95% CI 0.267–0.923, p = 0.027) (Table 3). Likewise, the association remained significant after adjusting for sex, age, BMI, and smoking habit (OR 0.503, 95% CI 0.269–0.941, p = 0.031), and even after adjusting for additional confounders including HbA1c, total cholesterol, HDL cholesterol, and triglycerides (OR 0.512, 95% CI 0.270–0.970, p = 0.040). When treatments and diabetes duration were further added to the fully adjusted logistic regression model the association between the C allele and lower risk of MI remained significant (OR 0.478, 95% CI 0.245–0.935, p = 0.031) (Table 3).

When a sensitivity analysis was carried out by excluding controls with coronary stenosis > 50% (i.e. considered as cases in the original GHS study-design), very similar results were obtained (Table 4).

**Table 4:** Sensitivity analysis of the association between rs9267551 and MI in the two study samples

Study samples	DDAH2 rs9267551 genotype			Additive model	
CZ (n = 1017)	GG	GC	CC	OR (95% CI)	p
MI-neg (n = 840)	757 (90.1%)	79 (9.4%)	4 (0.5%)	0.338 (0.144–0.793)	0.013^a^
				0.293 (0.101–0.850)	0.024^b^
MI-pos (n = 177)	170 (96.0%)	7 (4.0%)	0 (0%)	0.350 (0.113–1.0.82)	0.068^c^

GHS (n = 755)	GG	GC	CC	OR (95% CI)	p
M-neg (n = 571)	499 (87.4%)	69 (12.1%)	3 (0.5%)	0.479 (0.256–0.899)	0.022^a^
				0.487 (0.257–0.925)	0.028^b^
MI-pos (n = 184)	172 (93.5%)	12 (6.5%)	0 (0%)	0.454 (0.231–0.891)	0.022^c^

Pooled (n = 1772)	GG	GC	CC	OR (95% CI)	p
MI-neg (n = 1411)	1256 (89.0%)	148 (10.5%)	7 (0.5%)	0.429 (0.260–0.710)	0.001^d^
				0.434 (0.251–0.748)	0.003^e^
MI-pos (n = 361)	342 (94.7%)	19 (5.3%)	0 (0%)	0.432 (0.243–0.766)	0.004^f^

*CZ* Catanzaro Study, *GHS* Gargano Heart Study

^a^Adjusted for sex, age, BMI, smoking habit

^b^Adjusted for sex, age, BMI, smoking habit, HbA1c, total cholesterol, HDL cholesterol and triglycerides levels

^c^Adjusted for sex, age, BMI, smoking habit, HbA1c, total cholesterol, HDL cholesterol, triglycerides levels, diabetes duration and therapies

^d^Adjusted for sex, age, BMI, smoking habit and study center

^e^Adjusted for sex, age, BMI, smoking habit, HbA1c, total cholesterol, HDL cholesterol, triglycerides levels and study center

^f^Adjusted for sex, age, BMI, smoking habit, HbA1c, total cholesterol, HDL cholesterol, triglycerides levels, diabetes duration, therapies and study center

This makes very unlikely that changing the original GHS structure for the specific purpose of this investigation has played a role on the association we here report.

Since no significant evidence of heterogeneity was found between the two studies regarding the effect of rs9267551 polymorphism on the risk of MI (adjusted p for gene-by-sample interaction = 0.22), data from the two studies were pooled and jointly analyzed after adjusting for study sample. In the pooled analysis, the C allele was significantly associated with lower risk of MI (OR 0.458, 95% CI 0.283–0.739, p = 0.001) (Table 3). This association remained significant after adjusting for confounders including study center, gender, age, BMI, and smoking habit (OR 0.445, 95% CI 0.270–0.733, p = 0.001). The association remained significant after adjusting for additional confounders including HbA1c, total cholesterol, HDL cholesterol, and triglycerides (OR 0.458, 95% CI 0.266–0.787, p = 0.005), or when duration of diabetes and treatments were further added to the fully adjusted logistic regression model (OR 0.457, 95% CI 0.259–0.808, p = 0.007).

## Discussion

### State of the art

There is evidence that elevated levels of ADMA, an endogenous inhibitor of eNOS, are associated with CVD and mortality both in the general population as well as in individuals at high cardiovascular risk [7–10]. However, studies aimed at evaluating the association of ADMA with CVD in patients with T2DM has led to divergent results [12–15]. A possible explanation for these discrepancies could be due to the uncoupling of eNOS under hyperglycemic conditions [19, 28]. There is evidence that 5,6,7,8-tetrahydrobiopterin (BH4), an essential cofactor of eNOS, is oxidized in the setting of diabetes, resulting in impaired catalytic function, and uncoupling of eNOS that switches from nitric oxide (NO) to superoxide production [29]. In the presence of uncoupling of eNOS, such as in hyperglycemic conditions, ADMA-induced inhibition of eNOS activity may result in a reduction of superoxide production in the endothelium, and thus may be beneficial for the vasculature [28, 30]. On the other hand, an in vitro study has shown that ADMA is only a weak competitive inhibitor of eNOS, thus making the role of ADMA in vascular homeostasis even more complex, and only partially settled [30]. Additionally, the interpretation of results is further complicated by the findings that hyperglycemia may induce elevation in ADMA levels alongside with decreased ADMA degradation by DDAH [31]. Thus, whether elevated ADMA levels under diabetic milieu may reach a threshold capable to cause a significant inhibition in eNOS activity remains to be established. In an attempt to clarify this issue, we investigated the association of the rs9267551 polymorphism in the *DDAH2* gene with MI in T2DM patients taking advantage of the observation that this polymorphism has a functional impact with the minor C allele exhibiting a higher transcriptional activity resulting in enhanced *DDAH2* expression along with higher nitric oxide release in primary human endothelial cells [22]. In addition, the rs9267551 C allele has been associated with lower levels of circulating ADMA, higher insulin sensitivity assessed by euglycemic-hyperinsulinemic clamp studies, and lower risk of renal dysfunction [22, 32]. Interestingly, it has been shown that in C2C12 mouse myotubes ADMA is able to induce insulin resistance by reducing expression of both the insulin receptor substrate-1 and GLUT-4 glucose transporter, and increasing expression protein tyrosine phosphatase 1B (PTP1B), thus resulting in impaired insulin signaling, and reduced glucose uptake [33]. For these reasons, the rs9267551 polymorphism seems a plausible candidate in the search for variants in the *DDAH2* gene associated with MI amongst patients with T2DM.

### DDAH2 SNP and MI in T2DM

In the present study, we found that this polymorphism was significantly associated with MI in a total of 1839 T2DM patients of European ancestry from two independent cohorts with individuals carrying the C allele having a lower risk than carriers of the GG genotype. This association remained significant after adjustments for various cardiovascular risk factors including age, gender, BMI, smoking, duration of diabetes, and, lipid levels. Notably, the association remained also significant after adjustments for both glucose control assessed by HbA1c levels and anti-hypertensive and glucose-lowering treatments thus arguing against the possibility that factors affecting *DDAH2* expression or activity such as hyperglycemia, and treatments with angiotensin-converting enzyme (ACE) inhibitors, metformin, or pioglitazone, may explain the present results [31, 34–36]. The association remained also significant after adjustments for estimated glomerular filtration rate (eGFR) (data not shown), consistent with previous results showing that ADMA levels are not affected by glomerular filtration rates in diabetic subjects [37]. While GWAS data available for the general population [PhenoScanner database http://www.phenoscanner.medschl.cam.ac.uk/], suggested that the variant rs9267551 C was associated with lower risk of CAD (OR = 0.95, 95% CI 1.028–1.085; p = 3.88 × 10^−8^ [24]), to the best of our knowledge, this is the first study showing a role of the rs9267551 polymorphism in the *DDAH2* gene on MI in patients with T2DM. Because it has been reported that elevated levels of ADMA are associated with CVD in the general population and that they predicted mortality [7–10], it is possible that in subjects carrying the C allele less ADMA accumulates in endothelial cells causing vascular protection as a consequence of higher nitric oxide availability.

### Strengths

The present study has some strengths including the validation design with two independent cohorts of T2DM patients, the inclusion of both males and female, the homogeneity of the two patient samples recruited from two regions both from Central-Southern Italy, the exclusion of confounding conditions such as severe infectious illnesses or history of any malignant disease, the detailed characterization of patients by trained physicians in a clinical setting (no self-reported data were used) that allowed adjustment for several possible cardio-metabolic confounders, and the finding that formal replication of the association between rs9267551 polymorphism with MI reached nominal statistical significant in both the samples analyzed.

### Limitations

Nevertheless, this study must be interpreted within the context of its possible limitations. First, the cross-sectional design of the study allows to show only an association with prevalent, but not incident MI. Thus, although we were able to confirm in two cohorts the effect of the rs9267551 polymorphism on MI risk, the present results need replication in prospective studies before it can be considered as validated. Furthermore, measurements of circulating ADMA levels were not available and could not, therefore, be included in our analyses. However, we have previously shown that ADMA levels (measured by high performance liquid chromatography, i.e. the gold standard for ADMA concentration measurements) in non-diabetic individuals were significantly higher in sixty rs9267551 GG genotype carriers than in eight rs9267551 C allele carriers (0.68 ± 0.14 vs. 0.57 ± 0.14 µmol/l, respectively; p  =  0.04 after adjusting for age, gender, and BMI). An additional limitation of the study is represented by the robustness of our p-values which do not reach a genome-wide level of significance, and are, consequently, still compatible with a type I error (a false-positive result). Although the likelihood of such an event is reduced by the validation of the association in a publicly available database reporting GWAS data, and the hypothesis tested in this study is biologically plausible based on previous ‘‘in vitro’’ data [22], additional investigations are required before this association can be thought as fully established. Moreover, our study was limited to non-Hispanic White individuals, whether the present results can be extended to populations with different genetic background remains to be determined. Additionally, our cases were restricted to T2DM patients with MI, and whether the rs9267551 polymorphism is also associated with other CVD traits such as percutaneous transluminal coronary angioplasty or coronary artery bypass grafting, chronic ischemic heart disease, angina, and stroke is a subject worth exploring in future researches. Finally, residual confounding by unmeasured factors involved in control of DDAH expression and activity remains a possibility in this observational study.

## Conclusion

In conclusion, consistent with GWAS data observed in the general population (PhenoScanner database), the present results suggest a role of the functional polymorphism rs9267551 in the *DDAH2* gene in modulating the risk of MI among Italian patients with T2DM. Further studies are required to confirm these observations, and determine whether they can be extended to other populations of different ethnic background as well as to address the role of other variants in the *DDAH2* gene and their possible combined effect with that we herein reported.

## Data Availability

The dataset supporting the conclusions of this article is included within the article.

## Abbreviations

ADMA: asymmetric dimethylarginine
BMI: body mass index
CAD: coronary artery disease
CVD: cardiovascular disease
CZ: Catanzaro Study
DDAH: dimethylarginine dimethylaminohydrolase
eNOS: endothelial NO synthase
GHS: Gargano Heart Study-cross sectional design
GWAS: genome-wide association studies
HbA1c: glycated hemoglobin
MI: myocardial infarction
NO: nitric oxide
T2DM: type 2 diabetes
